# Oxygen: A Stimulus, Not “Only” a Drug

**DOI:** 10.3390/medicina57111161

**Published:** 2021-10-25

**Authors:** Costantino Balestra, Jacek Kot

**Affiliations:** 1 Laboratory of Environmental and Occupational (Integrative) Physiology, Haute Ecole Bruxelles-Brabant, Auderghem, 1160 Brussels, Belgium; costantinobalestra@gmail.com; 2 National Center of Hyperbaric Medicine in Gdynia, Medical University of Gdansk, 80-210 Gdansk, Poland

**Keywords:** oxygen, hyperbaric oxygen, epigenetics, normobaric oxygen paradox, hyperoxic-hypoxic paradox

## Abstract

Depending on the oxygen partial pressure in a tissue, the therapeutic effect of oxygenation can vary from simple substance substitution up to hyperbaric oxygenation when breathing hyperbaric oxygen at 2.5–3.0 ATA. Surprisingly, new data showed that it is not only the oxygen supply that matters as even a minimal increase in the partial pressure of oxygen is efficient in triggering cellular reactions by eliciting the production of hypoxia-inducible factors and heat-shock proteins. Moreover, it was shown that extreme environments could also interact with the genome; in fact, epigenetics appears to play a major role in extreme environments and exercise, especially when changes in oxygen partial pressure are involved. Hyperbaric oxygen therapy is, essentially, “intermittent oxygen” exposure. We must investigate hyperbaric oxygen with a new paradigm of treating oxygen as a potent stimulus of the molecular network of reactions.

## 1. Background

The usual path in medical sciences is typically the same: starting with understanding mechanisms; then conducting cellular tests using tissues, small animals, larger animals, then small human studies; and finally extensive clinical studies. Nevertheless, hyperbaric medicine has developed in an unusual way.

In fact, in the 1950s, a Dutch cardiac surgeon, Prof. Ite Boerema, began to use a hyperbaric chamber that was considered efficient in curing divers affected with the so-called “caissons’ disease” to help his newborn patients called “blue babies”. The procedure to mend cardiac septal defects needed a cardiac arrest, and the time for such a cardiac arrest was only minutes to secure a cardiovascular restart [1]. At that moment, an unacceptable number of patients were not surviving the surgery, and this surgeon wanted to find a method that would allow him to achieve better outcomes. His experiments were published in a famous paper entitled “Life without blood”, and he proved that it was possible to survive critical exsanguination while remaining in a hyperbaric chamber breathing high oxygen pressures (3.0 ATA).

Increasing circulating oxygen levels in the body by increasing the barometric pressure in the operating room permits survival even after a more prolonged cardiac arrest. Of course, some years later, extracorporeal circulation became available, and the “hyperbaric operating room” was no longer needed. Hyperbaric oxygen is no longer used for maintaining general oxygenation levels, and its use has been somewhat limited for the treatment of specific diseases that are not necessarily combined with hypoxemia. Since that very moment, many hyperbaric centers have been developed, and a long list of indications and procedures have been produced.

This medical field has been so “dispersive” that reactions arose, and hyperbaric medicine was even “coined” as a “therapy in search of diseases” [2].

Significant progress has been made since the 1960s, and investigations into the mechanisms that are underlying oxygen variations were awarded a Nobel prize in 2019 [3]. This is an essential milestone in the field as, today, we have sufficient understanding to tackle two significant points that still need to be investigated in hyperbaric medicine: how much and how often. Hyperbaric medicine should be called “oxygen medicine”; an extensive range of oxygen levels may be used; and, according to these levels, repetitions of sessions must be adapted.

## 2. The Challenge of Oxygen Partial Pressure

Depending on the oxygen partial pressure of tissues, the therapeutic effect of oxygenation can vary from simple substance substitution, when normobaric hyperoxia is used to restore tissue oxygen levels to normal values from (120 torr in arterial blood to approx. 30–40 torr in soft tissues), up to hyperbaric oxygenation when breathing of hyperbaric oxygen at 2.5–3.0 ATA gives tissue hyperoxia well above 1000 torr.

Interestingly, the dose-effect relation is not linear but is instead “U-shaped”. It is already the clinical standard that, at normobaric conditions, the arterial partial pressure must be kept in the relatively narrow range of 10–20 kPa (75–150 torr), as increased mortality was observed in critically ill patients exposed either to hypoxic or hyperoxic levels [4].

On the other hand, it is well known that exposing humans to hyperoxia induces oxygen toxicity (Paul Bert’s effect on the brain and the Lorrain–Smith effect on the lungs). 

## 3. The Challenge of Oxygen Toxicity

**Figure 1:**
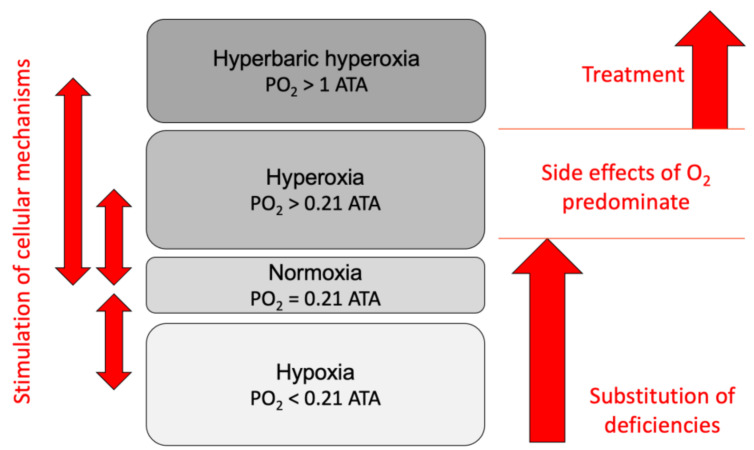
Oxygen levels and their therapeutic use (PO_2_, partial pressure of oxygen).

Today, the hyperbaric exposure dose (pressure and time) and repetitions are mainly limited by pulmonary and neurological toxicity. To remain on the safe side and not harm the patient, the OTUs (Oxygen Toxicity Units) or UTPDs (Units of Pulmonary Toxicity Dose) are calculated, even if those units are challenged in new approaches [5,6,7,8]. Permanent exposure to hyperbaric oxygen is not a clinical option. Fortunately, it seems that intermittent switching between low and high oxygen pressure is sufficiently potent to induce significant therapeutic molecular actions (Figure 1).

## 4. Oxygen as a Trigger

Surprisingly, new data show that even a minimal increase in the partial pressure of oxygen efficiently triggers cellular reactions [9,10]. As we already expressed in previous works, “some decades ago, on the physiological side, the two parameters that characterize extreme environments were identified as eliciting the production of two particular elements: hypoxia-inducible factors and heat-shock proteins. The two are ubiquitous and essential for cellular life” [11,12]. 

These “Hypoxia-Inducible Factors can trigger several hundred genes”, but it has been shown that hyperoxia, more specifically, coming back to normoxia after hyperoxic exposure (relative hypoxia), can trigger this essential factor responsible for vascular, cellular, and metabolic homeostasis and apoptosis [10,13,14]. Its beneficial actions in the fight against cancer cells have recently been advocated [12]. The second is a family of proteins acting as chaperones for other proteins and resetting impaired proteic structures triggered by many environmental stressors [15].

## 5. Epigenetics and the Challenge of Cellular Responses

Epigenetics seems to play a significant role in exercise and extreme environments [11]. Recently, it was shown that external stressors could indeed interact with the genome, especially when changes in the oxygen partial pressure are involved [16].

**Figure 2:**
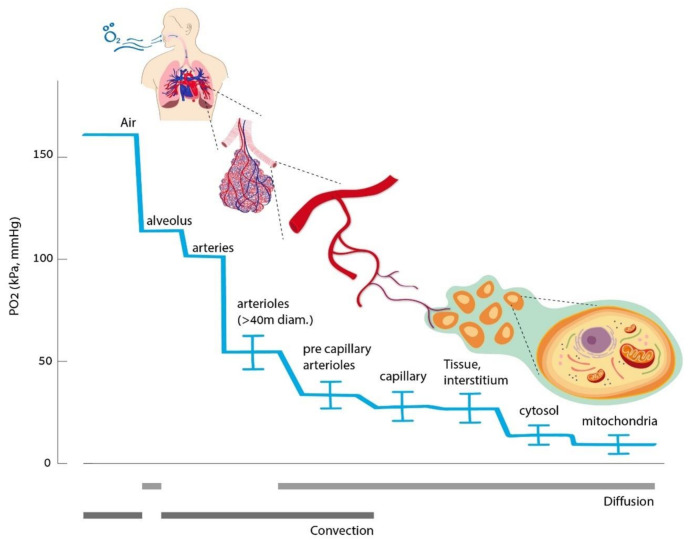
Oxygen cascade from ambient air down to mitochondria (figure taken from [17]).

This is may not be all that surprising as we know that physical exercise can produce extensive cellular reactions, including epigenetic reactions. Physical exercise is actually an intermittent oxidative stress variation. The oxygen level is very low in the mitochondria (Figure 2) [17]. We can, therefore, understand how potent a minute variation of oxygen tension may be at that level. We may need to consider oxygen variations in the mitochondrion as the most potent homeorhetic trigger in nature.

Considering this no wonder that even variations of 10% of the inspired oxygen fraction may be effective in humans and cultured cells [9,10,18]. It is now known that genes are not always activated. They are not mandatorily expressed. They can be “turned on” or “off” by external interferences that do not change the DNA sequence. There are two major mechanisms for this: DNA methylation and histone modifications. Acute environmental changes can induce epigenetic modifications; cells constantly receive all kinds of signals informing them about their surrounding environment and adjust their activity to the situation.

Recent data showed an epigenetic change through methylation in alpinists exposed to hypoxia, demonstrating rapid changes that were even recently not considered possible in the human epigenome following acute oxygen variation [16].

Several other articles have discussed the fact that pulsed hyperoxia induces hypoxia-inducible factor 1α (HIF-1α) activation and the expression of genes involved in the response to low oxygen describing a “normobaric oxygen paradox” (NOP), i.e., that relative changes of oxygen availability, rather than steady-state hypoxic (or hyperoxic) conditions, coordinate HIF-1α transcriptional effects [14,19]. This phenomenon has different names, either “Hyperoxic–Hypoxic Paradox” [17] or “Normobaric Oxygen Paradox” [20] depending on the range of variation of PO_2_ imposed. Nevertheless, a general term could be a “relative hypoxia” without reaching tissular hypoxic levels [20].

These studies investigated the activation of oxygen-sensitive transcription factors in peripheral blood mononuclear cells (PBMC) obtained from a human after breathing, increasing PO_2_, generating mild, high, and very high hyperoxia (30%, 100%, and 140% O_2_, respectively). The responses were followed for 24 h post exposure. It is possible that, for higher levels of hyperoxia (high and very-high), a longer time is needed to see reactive adaptations, such as the expression of nuclear factor erythroid 2-related factor 2 (NRF2) and HIF-1α. Further investigations are required with prolonged periods.

All treatments were associated with significant activation of NRF2 and HIF-1α. Conversely, the nuclear factor kappa B (NF-kB) transcription factor was significantly activated only by higher oxygen concentrations. The intracellular levels of total glutathione paralleled the nuclear transfer of NRF2 and remained elevated up to the end of the experimental observation time along with the plasmatic level of matrix metalloproteinase 9 (MMP-9). We confirmed that mild hyperoxia is sensed as hypoxic stress in vivo within the first 24 h, activating HIF-1α and NRF2, but not NF-kB.

Conversely, high hyperoxia was associated with a progressive loss of the NOP response and increased oxidative stress signals leading to NRF2 and NF-kB activation, accompanied by the synthesis of GSH. After very high hyperoxia, HIF-1α activation was absent in the first 24 h, and the oxidative stress response accompanied by NF-kB activation was prevalent. The glutathione (GSH) levels paralleled the nuclear transfer of NRF2 and remained elevated during the observation time together with the MMP-9 plasma levels. Further confirmation was published on the activation of microRNA during different oxygen exposures [21].

Interestingly, several articles have been interested in increasing hemoglobin and oxygen exposure [9,14,22,23,24]. To achieve such an increase in a fast way, small deltas were elicited; for a more extended response, higher oxygen variations and more distant (less frequent) variations were needed [21]. If the oxygen variations were relatively small, a repeated and frequent exposure had a fast answer (a few days) [22].

## 6. New Levels of Oxygen

Recent data confirmed that, in vivo, the return to normoxia after mild hyperoxia is sensed as hypoxic stress characterized by HIF-1α activation [10]. On the contrary, high hyperoxia and very high hyperoxia induce a shift toward an oxidative stress response, characterized by NRF2 and NF-κB activation in the first 24 h post-exposure.

Even though it is possible that higher levels of hyperoxia (high hyperoxia from 50% to 100%, very high hyperoxia at hyperbaric levels) may induce late responses to recover homeostasis over a longer window of time, previous studies in cultured cells [14] and in vivo [12,25] suggested that critical adaptive responses occur within shorter times. However, further investigations are needed to investigate if pulsed hyperoxia induces specific compensatory reactive adaptations at more extended periods. Future studies should focus on the two components of this paradigm: the oxygen exposure (time and PO_2_) and time between sessions (intermittent exposures) [12,23].

Overall, already published data [10] suggested the occurrence of a “hormetic” adaption to high oxygen triggered by the activation of signaling cascades leading to the expression of antioxidant systems; this is in agreement with data obtained in rodents undergoing either hyperbaric or normobaric oxygen that initially induced significant oxidative stress that was eventually resolved after continued exposure [26].

To optimize applicable clinical protocols from this paradigm, future studies are expected to focus also on the “down-stream” effects of HIF-1α transcriptional activation. Depending on the therapeutic target, using mild hyperoxia (from 30% to 40–50%) may be more desirable [12,27], or, on the other hand, eliciting oxidative stress utilizing HH/VHH administration may be considered a more appropriate desirable effect.

## 7. Intermittent Oxygen, the Clue?

Hyperbaric oxygen therapy is, by essence, “intermittent oxygen” exposure. It is clear from previous data that we must investigate this direction with a new paradigm of treating oxygen rather as a potent stimulus of the molecular network of reactions. The vital task is answering the questions of “how much”, “how long”, and “how often” this stimulus should be given. The non-linearity of the dose-response curve complicates the picture. Still, to the end, we should be able to answer what oxygen dose should be given to reach a specific clinical or molecular effect.

We hope that the authors of the papers in this special issue of *Medicina* and active readers using those ideas in their research will help to shape the future of “oxygen medicine”.

## References

[1] Boerema I., Meyne N.G., Brummelkamp W.H., Bouma S., Mensch M.H., Kamermans F., Stern Hanf M., van Aalderen W. (1960). Life without blood. Ned. Tijdschr. Geneeskd..

[2] Phillips T.J. (2000). Hyperbaric oxygen therapy: A therapy in search of a disease?. Dermatol. Surg..

[3] Lopez-Barneo J., Simon M.C. (2020). Cellular adaptation to oxygen deficiency beyond the Nobel award. Nat. Commun..

[4] De Jonge E., Peelen L., Keijzers P.J., Joore H., de Lange D., van der Voort P.H., Bosman R.J., de Waal R.A., Wesselink R., de Keizer N.F. (2008). Association between administered oxygen, arterial partial oxygen pressure and mortality in mechanically ventilated intensive care unit patients. Crit. Care.

[5] Wingelaar T.T., Brinkman P., Hoencamp R., van Ooij P.A., Maitland-van der Zee A.H., Hollmann M.W., van Hulst R.A. (2020). Assessment of pulmonary oxygen toxicity in special operations forces divers under operational circumstances using exhaled breath analysis. Diving Hyperb. Med..

[6] Arieli R., Aviner B. (2020). Acclimatization and Deacclimatization to Oxygen: Determining Exposure Limits to Avoid CNS O2 Toxicity in Active Diving. Front. Physiol..

[7] Aviner B., Arieli R., Yalov A. (2020). Power Equation for Predicting the Risk of Central Nervous System Oxygen Toxicity at Rest. Front. Physiol..

[8] Arieli R. (2020). Effect of an air break on the occurrence of seizures in hyperbaric oxygen therapy may be predicted by the power equation for hyperoxia at rest. Diving Hyperb. Med. J. S. Pac. Underw. Med. Soc..

[9] Khalife M., Ben Aziz M., Balestra C., Valsamis J., Sosnowski M. (2021). Physiological and Clinical Impact of Repeated Inhaled Oxygen Variation on Erythropoietin Levels in Patients After Surgery. Front. Physiol..

[10] Fratantonio D., Virgili F., Zucchi A., Lambrechts K., Latronico T., Lafere P., Germonpre P., Balestra C. (2021). Increasing Oxygen Partial Pressures Induce a Distinct Transcriptional Response in Human PBMC: A Pilot Study on the “Normobaric Oxygen Paradox”. Int. J. Mol. Sci..

[11] Balestra C., Kot J., Efrati S., Guerrero F., Blatteau J., Besnard S., Balestra C., Kot J., Efrati S., Guerrero F., Blatteau J., Besnard S. (2019). Coping with Extreme Environments: A Physiological/Psychological Approach.

[12] De Bels D., Corazza F., Germonpre P., Balestra C. (2011). The normobaric oxygen paradox: A novel way to administer oxygen as an adjuvant treatment for cancer?. Med. Hypotheses.

[13] De Bels D., Corazza F., Balestra C. (2011). Oxygen sensing, homeostasis, and disease. N. Engl. J. Med..

[14] Cimino F., Balestra C., Germonpre P., De Bels D., Tillmans F., Saija A., Speciale A., Virgili F. (2012). Pulsed high oxygen induces a hypoxic-like response in Human Umbilical Endothelial Cells (HUVECs) and in humans. J. Appl. Physiol..

[15] Blatteau J.E., Gempp E., Balestra C., Mets T., Germonpre P. (2008). Predive sauna and venous gas bubbles upon decompression from 400 kPa. Aviat. Space Environ. Med..

[16] Basang Z., Zhang S., Yang L., Quzong D., Li Y., Ma Y., Hao M., Pu W., Liu X., Xie H. (2021). Correlation of DNA methylation patterns to the phenotypic features of Tibetan elite alpinists in extreme hypoxia. J. Genet. Genom..

[17] Hadanny A., Efrati S. (2020). The Hyperoxic-Hypoxic Paradox. Biomolecules.

[18] Balestra C., Lambrechts K., Mrakic-Sposta S., Vezzoli A., Levenez M., Germonpré P., Virgili F., Bosco G., Lafère P. (2021). Hypoxic and Hyperoxic Breathing as a Complement to Low-Intensity Physical Exercise Programs: A Proof-of-Principle Study. Int. J. Mol. Sci..

[19] Balestra C., Germonpre P., Poortmans J.R., Marroni A. (2006). Serum erythropoietin levels in healthy humans after a short period of normobaric and hyperbaric oxygen breathing: The “normobaric oxygen paradox”. J. Appl. Physiol..

[20] Balestra C., Germonpre P. (2012). Hypoxia, a multifaceted phenomenon: The example of the “normobaric oxygen paradox”. Eur. J. Appl. Physiol..

[21] Bosco G., Paganini M., Giacon T.A., Oppio A., Vezzoli A., Dellanoce C., Moro T., Paoli A., Zanotti F., Zavan B. (2021). Oxidative Stress and Inflammation, MicroRNA, and Hemoglobin Variations after Administration of Oxygen at Different Pressures and Concentrations: A Randomized Trial. Int. J. Environ. Res. Public Health.

[22] Rocco M., D’Itri L., De Bels D., Corazza F., Balestra C. (2014). The “normobaric oxygen paradox”: A new tool for the anesthetist?. Minerva Anestesiol..

[23] Lafere P., Schubert T., De Bels D., Germonpre P., Balestra C. (2013). Can the normobaric oxygen paradox (NOP) increase reticulocyte count after traumatic hip surgery?. J. Clin. Anesth..

[24] De Bels D., Theunissen S., Devriendt J., Germonpre P., Lafere P., Valsamis J., Snoeck T., Meeus P., Balestra C. (2012). The ‘normobaric oxygen paradox’: Does it increase haemoglobin?. Diving Hyperb. Med. J. S. Pac. Underw. Med. Soc..

[25] Donati A., Damiani E., Zuccari S., Domizi R., Scorcella C., Girardis M., Giulietti A., Vignini A., Adrario E., Romano R. (2017). Effects of short-term hyperoxia on erythropoietin levels and microcirculation in critically Ill patients: A prospective observational pilot study. BMC Anesthesiol..

[26] Korpinar S., Uzun H. (2019). The Effects of Hyperbaric Oxygen at Different Pressures on Oxidative Stress and Antioxidant Status in Rats. Medicina.

[27] De Bels D., Tillmans F., Corazza F., Bizzari M., Germonpre P., Radermacher P., Orman K.G., Balestra C. (2020). Hyperoxia Alters Ultrastructure and Induces Apoptosis in Leukemia Cell Lines. Biomolecules.

